# Methotrexate is not associated with increased liver cirrhosis in a population-based cohort of rheumatoid arthritis patients with chronic hepatitis C

**DOI:** 10.1038/srep33104

**Published:** 2016-09-09

**Authors:** Kuo-Tung Tang, Yi-Hsing Chen, Ching-Heng Lin, Der-Yuan Chen

**Affiliations:** 1 Division of Allergy, Immunology and Rheumatology, Taichung Veterans General Hospital, Taichung, R.O.C; 2 Ph.D. Program in Translational Medicine, National Chung Hsing University, Taichung, R.O.C; 3 School of Medicine, National Yang-Ming University, Taipei, R.O.C; 4 Department of Medical Research, Taichung Veterans General Hospital, Taichung, R.O.C; 5 Institute of Microbiology and Immunology, Chung Shan Medical University, Taichung, R.O.C; 6 Institute of Biomedical Science, National Chung Hsing University, Taichung, R.O.C

## Abstract

A few studies have shown that methotrexate (MTX) use exacerbates liver fibrosis and even leads to liver cirrhosis in rheumatoid arthritis (RA) patients, although the risk is low compared to psoriatics. We therefore conducted a population-based cohort study to investigate the impact of long-term MTX use on the risk of chronic hepatitis C (CHC)-related cirrhosis among RA patients. We analyzed data from the National Health Insurance Research Database in Taiwan and identified 450 incident cases of RA among CHC patients (255 MTX users and 195 MTX non-users) from January 1, 1998 to December 31, 2007. After a median follow-up of more than 5 years since the diagnosis of CHC, a total of 55 (12%) patients developed liver cirrhosis. We did not find an increased risk of liver cirrhosis among CHC patients with long-term MTX use for RA. Furthermore, there was no occurrence of liver cirrhosis among the 43 MTX users with a cumulative dose ≧3 grams after 108 months of treatment. In conclusion, our data showed that long-term MTX use is not associated with an increased risk for liver cirrhosis among RA patients with CHC. However, these results should be interpreted with caution due to potential bias in the cohort.

Methotrexate (MTX) is commonly used for the treatment of autoimmune diseases such as rheumatoid arthritis (RA) and psoriasis^1^. There is controversy regarding the risk for liver cirrhosis in long-term MTX users. In general, the risk of MTX-related liver cirrhosis in RA patients seems to be lower than that in patients with psoriasis^2^. A prior meta-analysis concluded that 2.7% of RA patients would develop severe fibrosis or cirrhosis after 55 months of MTX treatment^3^. The results of our recent population-based study showed that long-term MTX use is not significantly associated with an increased risk for liver cirrhosis among RA patients with chronic hepatitis B (CHB)^4^.

Chronic hepatitis C (CHC) is one of the main etiological factors of liver cirrhosis^5^. In Taiwan, hepatitis C is endemic^6^. The impact of long-term MTX use on the risk of CHC-related cirrhosis among RA patients has not been investigated. Based on our previous findings^4^, we hypothesized that long-term MTX use does not increase the risk for liver cirrhosis among RA patients with CHC. We therefore conducted a retrospective cohort study based on the National Health Insurance Research Database (NHIRD) in Taiwan.

## Methods

### Patients

This retrospective cohort was based on the NHIRD^7^,^8^, which contains comprehensive healthcare claims data from more than 99% of the entire population of Taiwan (24 million people). In the database, diseases are coded with the International Classifications of Diseases, Ninth Revision, Clinical Modification (ICD-9-CM) codes. The diagnosis of RA (ICD-9-CM code 714.0) was made based on the 1987 ACR criteria^9^ using the Catastrophic Illness Patient Database (CIPD), a registry incorporating RA patients certified by two rheumatologists. The need for informed consent from individuals was waived as the NHIRD contains only de-identified data.

**Figure 1:**
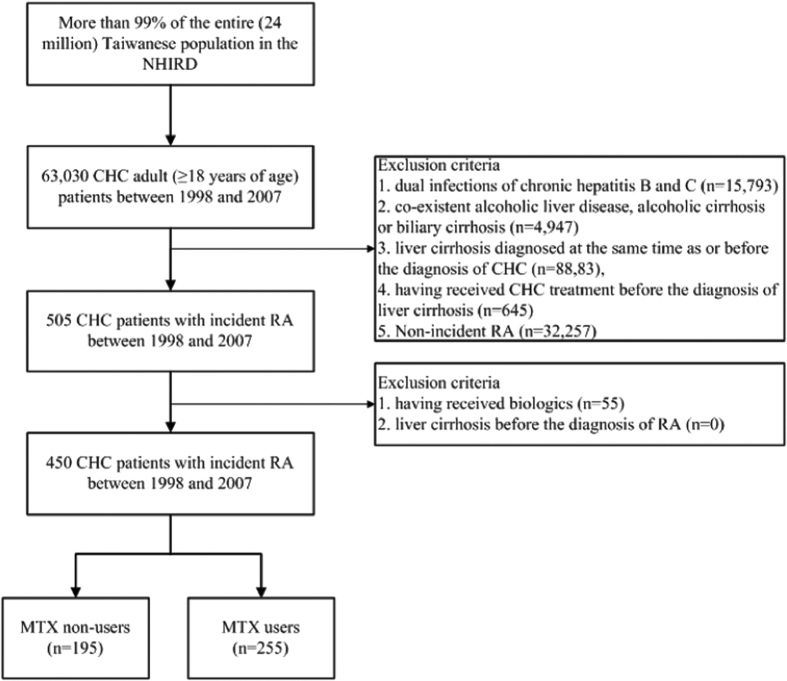
The flow chart of identification of RA patients with CHC. CHC, chronic hepatitis C; MTX, methotrexate; NHIRD, the National Health Insurance Research Database; RA, rheumatoid arthritis.

Figure 1 illustrates the study flowchart. We identified 63,030 adult patients who were diagnosed with CHC (ICD-CM codes 070.54, 070.70 or V02.62) in Taiwan from January 1, 1998 to December 31, 2007. Liver cirrhosis was defined as ICD-9-CM code 571.5. Exclusion criteria included dual infections of CHC and CHB (ICD-9-CM codes 070.2, 070.3 or V02.61), co-existent alcoholic liver disease (ICD-9-CM codes 571.0, 571.1 or 571.3), alcoholic cirrhosis (ICD-9-CM code 571.2) or biliary cirrhosis (ICD-9-CM code 571.6), pre-existing liver cirrhosis at the time of or before the diagnosis of CHC, and CHC treatment (ribavirin, peginterferon alfa-2b or peginterferon alfa-2a) before the diagnosis of liver cirrhosis. We then identified 505 incident RA cases among these CHC patients. Patients with liver cirrhosis before the diagnosis of RA or with biologics use (etanercept, adalimumab, golimumab, abatacept, tocilizumab, or rituximab) before the diagnosis of liver cirrhosis were also excluded. In total, 450 CHC patients with incident RA (consisting of 255 MTX users and 195 MTX non-users) were identified for analysis. This study was conducted in accordance with the Declaration of Helsinki and was approved by the Institutional Review Board of Taichung Veterans General Hospital (IRB TCVGH No.: CE15124B).

### Clinical parameters

Risk factors for liver cirrhosis, such as non-alcoholic fatty liver disease (NAFLD, ICD-9-CM code 571.8), diabetes mellitus (ICD-9-CM code 250), and dyslipidemia (ICD-9-CM code 272), as well as comorbidities were documented for each patient. Diagnosis of decompensated liver cirrhosis was made based on the inclusion in the CIPD. To be registered in the CIPD, at least one of the following criteria needs to be fulfilled for patients with liver cirrhosis: (1) intractable ascites, (2) variceal bleeding, or (3) hepatic coma or liver decompensation.

### Statistics

Statistical analysis was conducted using SAS software version 9.3 (SAS Institute, Cary, NC, USA.). All quantitative data are presented as means plus the standard deviation unless specified otherwise. For numerical variables, comparisons were made using the Student’s t test. For categorical variables, comparisons were made using the Chi-squared test. Hazard ratios of developing liver cirrhosis since the diagnosis of CHC between subgroups of RA patients were calculated using the Cox proportional hazards model after adjustment for age, gender, and comorbidities. The Kaplan-Meier survival analysis and the log rank test were used to compare the probability of liver cirrhosis-free survival since the diagnosis of CHC between MTX non-users and MTX users. A p value less than 0.001 in a two-sided test was considered statistically significant.

## Results

### Baseline characteristics of RA patients with CHC

Table 1 illustrates the baseline characteristics of RA patients with CHC. Leflunomide, sulfasalazine, and corticosteroids were prescribed more often in MTX users than in MTX non-users, indicating more severe disease activity of RA in MTX users compared with MTX non-users. In our 255 MTX users, the average cumulative MTX dosage was 1.6 ± 1.6 grams during a mean duration of 44 months. We categorized these MTX users into 3 groups based on the cumulative dose. The average durations of treatment were 18, 62, and 108 months; and the mean weekly doses were 8.3, 9.3, and 10.7 mg, within these groups of MTX users (MTX cumulative dose <1.5 grams, 1.5–3.0 grams, and ≧3.0 grams). After a median follow-up of more than 5 years since diagnosis of CHC, a total of 55 (12%) patients developed liver cirrhosis: 19 (7.5%) of 255 MTX users and 36 (18.5%) of 195 MTX non-users. Among the 19 MTX users who developed liver cirrhosis, 17 patients had a cumulative dose of ≦1.5 grams and 2 patients had a cumulative dose of ≧1.5 grams and <3.0 grams. One (0.4%) of 255 MTX users and 3 (1.5%) of 195 MTX non-users developed decompensated liver cirrhosis. Notably, there was no occurrence of liver cirrhosis among 43 MTX users with a cumulative dose of ≧3 grams.

### Multivariate analysis of risk factors for liver cirrhosis among RA patients with CHC

Table 2 illustrates the risk factors for liver cirrhosis in RA patients with CHC in the Cox proportional hazards model. Being older at diagnosis of CHC was a risk factor for liver cirrhosis. The concomitant use of MTX was associated with a decreased hazard of liver cirrhosis (hazard ratio: 0.33, 95% CI: 0.19–0.59), especially for MTX users with a cumulative dose of ≧1.5 grams (hazard ratio: 0.09, 95% CI: 0.02–0.37, Appendix 1), when compared with MTX non-users.

### Subgroup analysis of the incidence rates of liver cirrhosis among RA patients with CHC

Table 3 shows the subgroup analysis of the incidence rates of liver cirrhosis among RA patients with CHC. Among all patients, there was a trend toward a lower incidence rate of liver cirrhosis among MTX users compared with MTX non-users (11.2 versus 35.8 events per 1000 person-years, p = 0.004). We observed a trend toward a lower incidence rate of liver cirrhosis among MTX users when compared to MTX non-users among female patients, patients aged 45–64 years, and patients with concomitant use of sulfasalazine or corticosteroids.

### The probability of liver cirrhosis-free survival among RA patients with CHC

**Figure 2:**
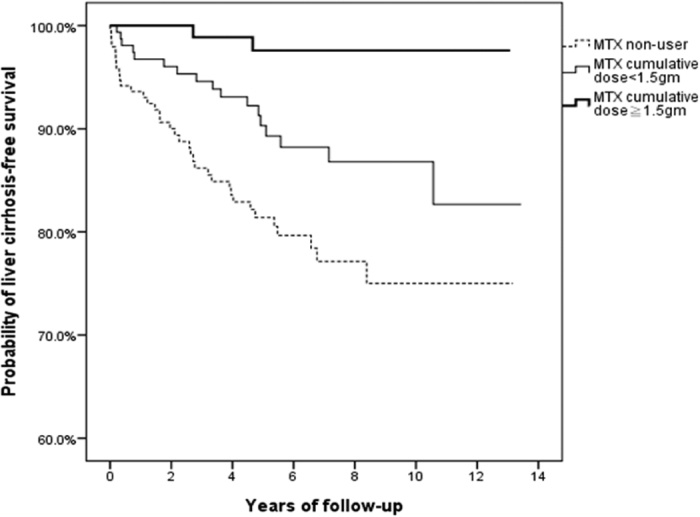
Probability of liver cirrhosis-free survival among MTX users with different cumulative doses and MTX non-users (p < 0.001, log-rank test). MTX, methotrexate.

Figure 2 illustrates the Kaplan-Meier survival analysis of liver cirrhosis-free survival with respect to MTX use among RA patients with CHC. The probability of liver cirrhosis-free survival was positively correlated with MTX use in a dose-dependent manner: the highest probability was in MTX users with a cumulative dose of ≧1.5 grams, and the lowest probability was in MTX non-users (p < 0.001, log-rank test).

## Discussion

This population-based cohort study investigated the impact of long-term MTX use on the risk of liver cirrhosis among RA patients with CHC. There was no significant increase of liver cirrhosis in RA patients with CHC who received long-term MTX treatment, which was in line with our previous results in RA patients with CHB^4^. However, no firm conclusion can be made due to potential bias in the cohort.

Among RA patients with CHC in Taiwan, 255 (57%) of 455 patients (excluding those patients who had received biologics) had received MTX to treat RA, a prevalence comparable to that among RA patients with CHB^4^. Therefore, MTX was still used by over half of the RA patients with chronic viral hepatitis in an endemic area, reflecting its fundamental role in RA treatment^10^. In terms of comorbidities, the prevalence of NAFLD in our RA patients was lower than rates reported in earlier studies that described liver biopsy findings in RA patients^11^,^12^, probably due to suboptimal monitoring of RA patients for comorbidities^13^.

After a median follow-up of more than 5 years since the diagnosis of CHC, 55 (12%) of 450 RA patients with CHC developed liver cirrhosis. This incidence of liver cirrhosis was higher than among RA patients with CHB (6.5%, p < 0.001)^4^. With respect to the risk for liver cirrhosis, there are no studies directly comparing CHB and CHC patients, and indirect comparisons remain inconclusive due to wide variations in the risk for liver cirrhosis among patients with chronic viral hepatitis^14^,^15^. In addition, our RA patients with CHC were slightly older than patients with CHB^4^, which increased the risk of liver cirrhosis^15^,^16^. Further studies are needed to elucidate this issue.

Previous studies have demonstrated that the risk for liver cirrhosis after long-term MTX therapy is low among RA patients^3^. Our recent study also demonstrated that long-term MTX use is not associated with an increased risk for liver cirrhosis even among RA patients with a comorbidity that contributes to liver cirrhosis, such as CHB^4^. In the present study, a significantly lower proportion of MTX users developed liver cirrhosis than MTX non-users (8% vs. 19%, p < 0.001), although MTX users were followed for a longer period than non-users (6.5 vs. 5.2 years, p < 0.001). In the Cox proportional hazards model, being older at CHC diagnosis, as reported in a previous study^15^, was identified as a significant risk factor for developing liver cirrhosis among RA patients with CHC, whereas MTX use was inversely associated with the development of liver cirrhosis among RA patients with CHC. Moreover, we found a significant increase in liver cirrhosis-free survival among MTX users in a dose-dependent manner when compared to MTX non-users, although higher proportions of concomitant use of hepatotoxic drugs such as sulfasalazine and leflunomide^1^, and corticosteroids, which are associated with increased hepatitis C RNA titers^17^, were found in MTX users. In addition, there was no occurrence of liver cirrhosis among 43 MTX users with a cumulative dose of ≧3 grams after 108 months of treatment, a finding similar to that observed among RA patients with CHB^4^. Taken together, these findings indicate that long-term MTX use was not associated with an increased risk, but was even associated with a decreased risk of liver cirrhosis among RA patients with CHC.

However, it is not reasonable to infer that MTX use is protective against liver cirrhosis due to the well-known hepatoxicity of MTX^18^. Rather, some other characteristics alleviate the risk for liver cirrhosis among RA patients with CHC who used MTX. One of the possible explanations might be obesity. In a study enrolling 24 patients on long-term MTX therapy for psoriasis, serial liver biopsies revealed that non-alcoholic steatohepatitis (NASH) is an important cause of liver injury in such patients, and a high cumulative dose of MTX alone can lead to a NASH-like liver injury pattern^19^. This observation was corroborated by a recent analysis of individuals who received or had been listed for liver transplantation in the US, which shows that the risk factor profile of end-stage MTX-related liver disease is similar to that of NASH^20^. In our recent study of RA patients with CHB^4^, we speculated that compared to MTX users, MTX non-users had less severe disease activity of RA, which was associated with higher body mass index^21^,^22^. This was correlated with the development of NAFLD^23^, which contributed to the development of liver cirrhosis in MTX non-users among RA patients with CHB^23^. This postulate might also explain, at least in part, the results of the present study. However, one may argue that there was no difference in the prevalence of diagnosed NAFLD between MTX users and non-users in the present study. Nonetheless, the prevalence of NAFLD might have been underestimated due to the reason described above.

Our study has some limitations. First, as mentioned in our previous study^4^, a cohort study based on a claims database is potentially biased despite efforts to adjust for confounding variables. Nevertheless, an analysis using a real-world population-based database can still provide important insights. Second, a longer follow-up with a higher cumulative MTX dose may be needed to observe the contribution of MTX to the risk of HCV-related liver cirrhosis. Third, the medication effect might have been underestimated due to nonadherence. Fourth, anthropometric measurements are not available in the NHIRD, and, therefore, we could not evaluate the impact of obesity. Finally, daily alcohol consumption, a risk factor for HCV-related liver cirrhosis, is not documented in the NHIRD. However, we excluded patients with co-existent alcoholic liver disease and/or alcoholic cirrhosis in the cohort, and such bias is minimized.

In conclusion, our data showed that long-term MTX use is not associated with an increased risk for liver cirrhosis among RA patients with a comorbidity that contributes to liver cirrhosis, such as CHC. This finding is compatible with our previous observation in RA patients with CHB. However, the results should be interpreted with caution due to potential bias in the cohort.

## Additional Information

**How to cite this article**: Tang, K.-T. *et al.* Methotrexate is not associated with increased liver cirrhosis in a population-based cohort of rheumatoid arthritis patients with chronic hepatitis C. *Sci. Rep.* **6**, 33104; doi: 10.1038/srep33104 (2016).

## Supplementary Material

Supplementary Information

## Figures and Tables

**Table 1:** Baseline characteristics of rheumatoid arthritis patients with CHC.

Variables	MTX users (n = 255)	MTX nonusers (n = 195)
Age at diagnosis of CHC (years)	57.7 ± 12.7	61.0 ± 13.3
Gender		
Female	205 (80%)	151 (77%)
Male	50 (20%)	44 (23%)
Comorbidity		
NAFLD	7 (3%)	0 (0%)
Diabetes mellitus	46 (18%)	33 (17%)
Dyslipidemia	41 (16%)	37 (19%)
Hypertension	82 (32%)	86 (44%)
Concomitant medications		
Leflunomide	35 (14%)	5 (3%)*
Azathioprine	11 (4%)	3 (2%)
Sulfasalazine	152 (60%)	68 (35%)*
Corticosteroids	205 (80%)	92 (47%)*
MTX		
Cumulative dose <1.5 grams	163 (64%)	NA
Cumulative dose 1.5–3.0 grams	49 (19%)	NA
Cumulative dose ≧3.0 grams	43 (17%)	NA
Liver cirrhosis	19 (8%)	36 (19%)*
Decompensated liver cirrhosis	1 (0%)	3 (2%)
Median follow-up period after the diagnosis of CHC (years)	6.5	5.2*

^*^p < 0.001, versus MTX users.

CHC: chronic hepatitis C; MTX: methotrexate; NA: not avalaible; NAFLD: non-alcoholic fatty liver disease.

**Table 2:** Multivariate analysis for liver cirrhosis among CHC patients with rheumatoid arthritis.

Variables	Adjusted HR (95% CI)
Age at diagnosis of CHC (years)	1.05 (1.03–1.08)**
Gender	
Female	1.00
Male	1.13 (0.59–2.17)
MTX nonusers	1.00
MTX users	0.33 (0.19–0.59)**
Comorbidity	
Diabetes mellitus	2.71 (1.44–5.11)*
Dyslipidemia	0.75 (0.35–1.59)
Hypertension	0.54 (0.30–0.97)*

*p < 0.05; **p < 0.001.

CHC: chronic hepatitis C; CI: confidence interval; MTX: methotrexate; NAFLD: non-alcoholic fatty liver disease.

**Table 3:** Subgroup analysis for new-onset liver cirrhosis in rheumatoid arthritis patients with CHC.

Variables	MTX users	MTX non-users	Adjusted hazard ratio (95% CI)#
	Incidence rate of liver cirrhosis (per 1,000 person-years)	Incidence rate of liver cirrhosis (per 1,000 person-years)	
All patients	11.2	35.8	0.40 (0.21–0.74)*
Age at the diagnosis of CHC (years)			
18–44	0.0	6.3	NA
45–64	8.7	32.2	0.34 (0.13–0.91)*
≧65	24.6	56.7	0.50 (0.22–1.12)
Gender			
Female	10.8	33.8	0.44 (0.21–0.89)*
Male	13.3	45.2	0.38 (0.10–1.55)
Comorbidity			
Diabetes mellitus	21.3	95.2	0.32 (0.09–1.15)
Dyslipidemia	11.3	40.3	0.57 (0.11–3.07)
Hypertension	13.4	29.2	0.66 (0.22–1.98)
Concomitant medications			
Sulfasalazine	8.3	25.4	0.38 (0.15–0.99)*
Corticosteroids	8.7	32.8	0.27 (0.12–0.61)*

^*^p < 0.05.

CHC: chronic hepatitis C; CI: confidence interval; MTX: methotrexate; NA: not available.

^#^Adjusted for age, gender, diabetes mellitus, dyslipidemia, hypertension and medications such as sulfasalazine and corticosteroids.

## References

[b1] Aithal G. P. Hepatotoxicity related to antirheumatic drugs. Nat Rev Rheumatol 7, 139–150 (2011). 21263458 10.1038/nrrheum.2010.214

[b2] Zachariae H. Liver biopsies and methotrexate: a time for reconsideration? J Am Acad Dermatol 42, 531–534 (2000). 10688735 10.1016/s0190-9622(00)90237-8

[b3] Whiting-O’Keefe Q. E., Fye K. H. & Sack K. D. Methotrexate and histologic hepatic abnormalities: a meta-analysis. Am J Med 90, 711–716 (1991). 1828327

[b4] Tang K. T., Hung W. T., Chen Y. H., Lin C. H. & Chen D. Y. Methotrexate is not associated with increased liver cirrhosis in a population-based cohort of rheumatoid arthritis patients with chronic hepatitis B. Sci Rep 6, 22387 (2016). 26928373 10.1038/srep22387 PMC4772158

[b5] Heidelbaugh J. J. & Sherbondy M. Cirrhosis and chronic liver failure: part II. Complications and treatment. Am Fam Physician 74, 767–776 (2006). 16970020

[b6] Chen D. S. *et al.* Hepatitis C virus infection in an area hyperendemic for hepatitis B and chronic liver disease: the Taiwan experience. J Infect Dis 162, 817–822 (1990). 2169497 10.1093/infdis/162.4.817

[b7] Huang S. W. *et al.* Osteoarthritis increases the risk of dementia: a nationwide cohort study in Taiwan. Sci Rep 5, 10145 (2015). 25984812 10.1038/srep10145 PMC4434986

[b8] Hung C. Y. *et al.* Age and CHADS2 Score Predict the Effectiveness of Renin-Angiotensin System Blockers on Primary Prevention of Atrial Fibrillation. Sci Rep 5, 11442 (2015). 26094981 10.1038/srep11442 PMC4476091

[b9] Arnett F. C. *et al.* The American Rheumatism Association 1987 revised criteria for the classification of rheumatoid arthritis. Arthritis Rheum 31, 315–324 (1988). 3358796 10.1002/art.1780310302

[b10] Jacobs J. W. Lessons for the use of non-biologic anchor treatments for rheumatoid arthritis in the era of biologic therapies. Rheumatology (Oxford) 51 Suppl 4, iv27–iv33 (2012). 22685273 10.1093/rheumatology/kes084

[b11] Dietrichson O., From A., Christoffersen P. & Juhl E. Morphological changes in liver biopsies from patients with rheumatoid arthritis. Scand J Rheumatol 5, 65–69 (1976). 935824 10.3109/03009747609099892

[b12] Rau R., Pfenninger K. & Boni A. Proceedings: Liver function tests and liver biopsies in patients with rheumatoid arthritis. Ann Rheum Dis 34, 198–199 (1975). 1137451 10.1136/ard.34.2.198-d PMC1006382

[b13] Dougados M. *et al.* Prevalence of comorbidities in rheumatoid arthritis and evaluation of their monitoring: results of an international, cross-sectional study (COMORA). Ann Rheum Dis 73, 62–68 (2014). 24095940 10.1136/annrheumdis-2013-204223 PMC3888623

[b14] Fattovich G., Bortolotti F. & Donato F. Natural history of chronic hepatitis B: Special emphasis on disease progression and prognostic factors. Journal of Hepatology 48, 335–352 (2008). 18096267 10.1016/j.jhep.2007.11.011

[b15] Thein H. H., Yi Q., Dore G. J. & Krahn M. D. Estimation of stage-specific fibrosis progression rates in chronic hepatitis C virus infection: a meta-analysis and meta-regression. Hepatology 48, 418–431 (2008). 18563841 10.1002/hep.22375

[b16] Chen C. J. & Yang H. I. Natural history of chronic hepatitis B REVEALed. J Gastroenterol Hepatol 26, 628–638 (2011). 21323729 10.1111/j.1440-1746.2011.06695.x

[b17] Henry S. D., Metselaar H. J., Van Dijck J., Tilanus H. W. & Van Der Laan L. J. Impact of steroids on hepatitis C virus replication *in vivo* and *in vitro*. Ann N Y Acad Sci 1110, 439–447 (2007). 17911459 10.1196/annals.1423.046

[b18] Salliot C. & van der Heijde D. Long-term safety of methotrexate monotherapy in patients with rheumatoid arthritis: a systematic literature research. Ann Rheum Dis 68, 1100–1104 (2009). 19060002 10.1136/ard.2008.093690 PMC2689525

[b19] Langman G., Hall P. M. & Todd G. Role of non-alcoholic steatohepatitis in methotrexate-induced liver injury. J Gastroenterol Hepatol 16, 1395–1401 (2001). 11851839 10.1046/j.1440-1746.2001.02644.x

[b20] Dawwas M. F. & Aithal G. P. End-stage methotrexate-related liver disease is rare and associated with features of the metabolic syndrome. Aliment Pharmacol Ther 40, 938–948 (2014). 25185870 10.1111/apt.12912

[b21] Fleming A., Crown J. M. & Corbett M. Prognostic value of early features in rheumatoid disease. Br Med J 1, 1243–1245 (1976). 1083760 10.1136/bmj.1.6020.1243 PMC1639812

[b22] Kaufmann J., Kielstein V., Kilian S., Stein G. & Hein G. Relation between body mass index and radiological progression in patients with rheumatoid arthritis. J Rheumatol 30, 2350–2355 (2003). 14677176

[b23] Chalasani N. *et al.* The diagnosis and management of non-alcoholic fatty liver disease: practice Guideline by the American Association for the Study of Liver Diseases, American College of Gastroenterology, and the American Gastroenterological Association. Hepatology 55, 2005–2023 (2012). 22488764 10.1002/hep.25762

