# Supplementary material for: Methotrexate is not associated with increased liver cirrhosis in a population-based cohort of rheumatoid arthritis patients with chronic hepatitis C
Source: Sci Rep. 2016 Sep 9;6:33104. doi: 10.1038/srep33104 (PMC5016832; doi:10.1038/srep33104)
Supplement: Supplementary Information [file srep33104-s1.doc]

**Title: Methotrexate is not associated with increased liver cirrhosis in a population-based cohort of rheumatoid arthritis patients with chronic hepatitis C.**

Kuo-Tung Tang, Yi-Hsing Chen, [Ching-Heng Lin](http://rheumatology.oxfordjournals.org/search?author1=Ching-Heng+Lin&sortspec=date&submit=Submit), Der-Yuan Chen

**Appendix 1.** Incorporating subgroups of MTX users with different

cumulative doses in the multivariate analysis for liver cirrhosis in

rheumatoid arthritis patients with chronic hepatitis C.

| Variables | Adjusted HR (95% CI) |
| --- | --- |
| Age at diagnosis of chronic hepatitis C (years) | 1.05 (1.03-1.08)** |
| Gender |  |
| Female | 1.00 |
| Male | 1.05 (0.55-2.03) |
| MTX nonusers | 1.00 |
| MTX users |  |
| MTX cumulative dose < 1.5 grams | 0.50 (0.27-0.90)* |
| MTX cumulative dose ≧ 1.5 grams | 0.09 (0.02-0.37)* |
| Comorbidity |  |
| Diabetes mellitus | 2.65 (1.39-5.04)* |
| Dyslipidemia | 0.66 (0.31-1.42) |
| Hypertension | 0.53 (0.30-0.95)* |

*p<0.05; **p<0.001

CI: confidence interval; MTX: methotrexate; NAFLD:

non-alcoholic fatty liver disease
